# Extracellular Vesicles from Monocyte-Derived Dendritic Cells Modulate Lymphocyte and Eosinophil Responses in Cow’s Milk Allergy

**DOI:** 10.3390/ijms27041977

**Published:** 2026-02-19

**Authors:** Antonio Serrano-Santiago, Daniel Rodríguez-González, Gema Guillén-Sánchez, Álvaro Arranz-Fragua, Rebeca López-Gómez, Ana Ladrón-Guevara, Rosa María Luz-Romero, Raquel Mirasierra-Pérez, Genoveva del Río Camacho, Victoria del Pozo, José Antonio Cañas

**Affiliations:** 1 Immunology Department, Health Research Institute-Fundación Jiménez Díaz, Universidad Autónoma de Madrid (IIS-FJD, UAM), 28040 Madrid, Spain; antonio.serrano.santiago@gmail.com (A.S.-S.); daniel.rodriguezg02@estudiante.uam.es (D.R.-G.); gema.gsanchez@quironsalud.es (G.G.-S.); alvaro.arranzf@estudiante.uam.es (Á.A.-F.); vpozo@iis-fjd.es (V.d.P.); 2 Centro de Investigación Biomédica en Red (CIBER) de Enfermedades Respiratorias (CIBERES), 28029 Madrid, Spain; 3 Pediatrics Department, University Hospital Fundación Jiménez Díaz, 28040 Madrid, Spain; rebeca.lopezg@quironsalud.es (R.L.-G.); ana.ladron@quironsalud.es (A.L.-G.); rosa.luz@quironsalud.es (R.M.L.-R.); raquel.mirasierra@quironsalud.es (R.M.-P.); grcamacho@fjd.es (G.d.R.C.); 4 Medicine Department, School of Medicine, Faculty of Medicine, Universidad Autónoma de Madrid (UAM), 28029 Madrid, Spain

**Keywords:** cow’s milk allergy, extracellular vesicles, monocyte-derived dendritic cells, eosinophils, lymphocytes, IgE-mediated

## Abstract

Cow’s milk allergy (CMA) is characterized by an exaggerated immune response where dendritic cells (DCs) play a crucial role. Additionally, extracellular vesicles (EVs) can be released by immune cells, modulating this allergic response. Moreover, eosinophils also contribute to tissue damage and perpetuate inflammation in allergic reactions. Therefore, the aim of this work was to study the role of EVs from monocyte-derived dendritic cells (moDCs) on eosinophil and lymphocytes in CMA. Sixteen infants with IgE-mediated cow’s milk allergy (CMAIE) and three non-allergic controls were recruited. Peripheral blood monocytes were purified and differentiated to moDCs. EVs were obtained from the culture supernatant by ultracentrifugation and characterized by nanoparticle tracking analysis and Western blot. Interaction among EVs, eosinophils and peripheral blood mononuclear cells (PBMCs) were analyzed with confocal microscopy. Additionally, these cells were incubated with EVs to assess lymphocyte proliferation, as well as eosinophil migration and reactive oxygen species (ROS) production by flow cytometry. Moreover, multiplex analysis was performed to evaluate the cytokines released by PBMCs following stimulation with EVs. Proteins characteristic of EVs were identified (CD9, CD63, CD81 and Alix). Furthermore, the size of the nanovesicles was ~185 nm, which is consistent with previously published reports. Confocal microscopy revealed that EVs internalized and localized in the cytoplasm of eosinophils, while in PBMCs, EVs were located in the perinuclear region. A proliferation assay revealed an increase in the proliferation of Th1 and Th2 lymphocytes, with higher levels of IL-4. Moreover, EVs were able to significantly increase eosinophil ROS production and migration. However, these effects were not observed after stimulation with EVs from non-allergic controls. This exploratory study shows that EVs from the moDCs of children with CMAIE could induce chemotactic and stimulatory functions on eosinophils and lymphocytes, which could perpetuate inflammation and contribute to tissue damage in this type of allergy.

## 1. Introduction

Allergic diseases, particularly food allergies, have markedly increased in prevalence over recent decades, becoming a major global health concern. These disorders result from a complex interplay between genetic predisposition and environmental influences. Specifically, cow’s milk allergy (CMA) stands out as one of the most common food allergies in early childhood, with symptoms typically emerging within the first year of life [1,2]. CMA is an immune-mediated hypersensitivity reaction triggered by specific cow’s milk proteins, mainly caseins (αs1-, αs2-, β-, and κ-casein) and whey proteins (α-lactalbumin, β-lactoglobulin, and bovine serum albumin) [3].

From a nutritional perspective, cow’s milk is a fundamental staple in the infant diet, providing a dense source of high-quality proteins, calcium, and essential vitamins required for rapid growth and bone development [4]. Consequently, the current standard of care, strict dietary avoidance, poses significant nutritional challenges. Prolonged exclusion of dairy without appropriate substitution can lead to growth faltering and micronutrient deficiencies [5]. While extensively hydrolyzed formulas (eHF) and amino acid-based formulas (AAF) are effective clinical alternatives, they are often limited by high costs and poor palatability. Furthermore, the increasing use of plant-based beverages (e.g., rice, almond, or soy) as unfortified substitutes raises concerns regarding their nutritional adequacy for infants [4,6]. Therefore, understanding the underlying cellular mechanisms of CMA is not only critical for immunological intervention but also for developing strategies to restore oral tolerance, thereby ensuring optimal nutritional status.

CMA involves aberrant immune activation that may be IgE-mediated, non-IgE-mediated, or mixed [7]. Clinical manifestations range from cutaneous and gastrointestinal symptoms to potentially life-threatening anaphylaxis, significantly impacting nutritional status and quality of life in infants [8]. CMA affects 2–6% of infants, with IgE-mediated mechanisms accounting for about 60% of pediatric cases [9]. Most children develop tolerance by the age of five, though 10–20% remain allergic into adulthood [10]. The prevalence is notably higher in industrialized countries, likely due to factors such as reduced microbial exposure, dietary changes, and urbanization [11].

At the cellular and molecular levels, CMA can be classified into IgE-mediated, non-IgE-mediated or mixed reactions [12]. Specifically, in IgE-mediated CMA (CMAIE) reactions, Th2 polarization promotes B-cell production of allergen-specific IgE antibodies [13]. Mixed reactions can also occur, involving both IgE and non-IgE pathways.

Dendritic cells (DCs) are professional antigen-presenting cells that are key elements in the initiation and regulation of allergic reactions. Beyond their classical functions, DCs release extracellular vesicles (EVs) that can modulate these allergic reactions [14,15]. EVs are membrane-enclosed nanoparticles secreted by almost all cell types into the extracellular space, functioning as versatile mediators of intercellular communication [16]. They carry a diverse array of bioactive molecules, including proteins, nucleic acids (DNA, RNA, and microRNAs [miRNAs]), and lipids that enable them to regulate various biological processes such as immune responses, tumor progression, and the propagation of neurodegenerative diseases [17]. Based on their biogenesis pathways, EVs are commonly divided into three main subtypes: exosomes (50–150 nm), ectosomes or microvesicles (100 nm to several micrometers), and apoptotic bodies (1–5 μm) [18]. These nanovesicles carry immunomodulatory molecules including MHC I/II, CD40, CD80, CD86, ICAM-1, tetraspanins (CD9, CD63, CD81), and microRNAs [16], participating actively in intercellular communication. In allergic diseases such as asthma and rhinitis, EVs have been shown to promote Th2 polarization of CD4^+^ T cells, amplifying allergic inflammation [19]. Given the pivotal role of DCs in orchestrating allergic immune responses and the emerging evidence that EVs serve as potent mediators of intercellular communication, investigating DC-derived EVs offers a promising avenue to uncover novel mechanisms of immune modulation in CMA.

Therefore, in this study, we explored the role of EVs derived from monocyte-derived DCs (moDCs) obtained from CMAIE infants. Specifically, we examined how these EVs influence eosinophil migration and reactive oxygen species (ROS) production, as well as T lymphocyte proliferation and cytokine release, to gain deeper insight into the cellular and molecular interactions driving allergic inflammation in CMA. Additionally, this opens a therapeutic avenue, as targeting EV biogenesis may represent a promising strategy for allergy management. Experimental inhibition of EV release could modulate T-cell and eosinophil responses, highlighting the potential of EV-based interventions in CMA.

## 2. Results

### 2.1. Study Subjects and Clinical Data

A cohort of infants aged 0–12 months, including 16 infants with CMAIE and three non-allergic controls, was recruited. Both groups were comparable in terms of demographic variables, including sex distribution and age at recruitment.

Clinical manifestations in the CMAIE group were consistent with immediate-type hypersensitivity reactions following exposure to cow’s milk proteins. All affected infants presented cutaneous symptoms; six patients (37%) exhibited gastrointestinal manifestations; and two infants (12%) showed respiratory symptoms. As expected, serological evaluation revealed a marked elevation of total and specific IgE levels (α-lactalbumin, β-lactoglobulin, and casein) in CMAIE patients compared with controls (Table 1).

These observations confirm that the patient cohort accurately represents the typical immunophenotype and clinical presentation of IgE-mediated cow’s milk allergy in infancy.

### 2.2. moDC-Derived Extracellular Vesicles Display Typical Size Distribution and EVs Marker Profile

**Figure 1:**
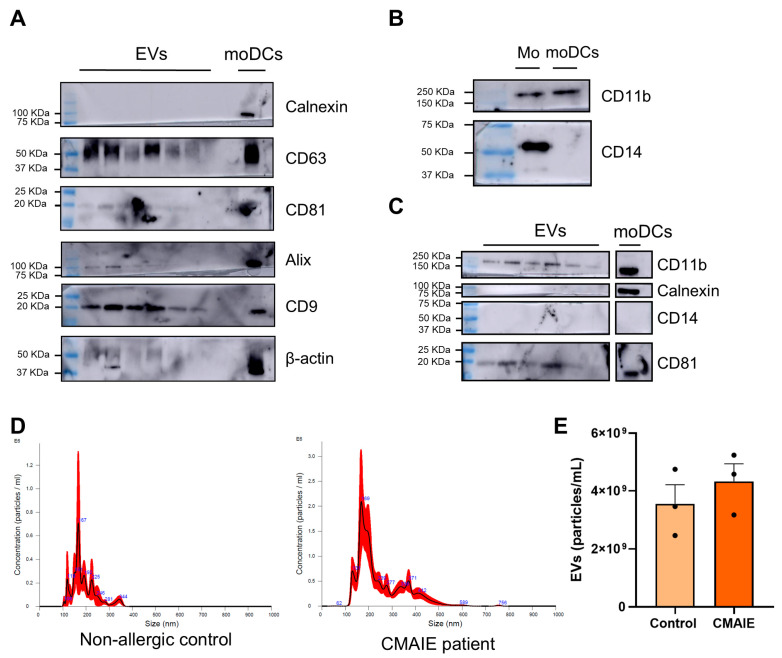
**Characterization of EVs released by moDCs.** (**A**) Immunoreactive bands for EV-specific markers—CD81, CD9, CD63, and ALIX—were observed. Β-actin and calnexin bands were not observed in EV samples. (**B**) Immunoreactive bands linked to CD14 (60 kDa) and CD11b (170 kDa) were observed in samples from monocytes and/or moCDs. (**C**) Immunoreactive bands corresponding to CD11b (170 kDa), calnexin (90 kDa) and CD81 (22 kDA). No immunoreactivity was observed for CD14 in moDCs. (**D**) Representative size distribution profiles of EVs derived from non-allergic controls and CMAIE patients. (**E**) Concentration of EVs from three independent samples obtained from both the control and CMAIE groups. CMAIE: children with IgE-mediated cow’s milk allergy. EVs: extracellular vesicles, moDCs: monocyte-derived dendritic cells, Mo: monocytes.

To ensure the identity and purity of the EVs released by moDCs, a phenotypic characterization was performed (Figure 1) according to several recommendations of the Minimal Information for Studies of Extracellular Vesicles (MISEV2023) guidelines [20]. Western blot analysis confirmed the presence of immunoreactive bands for EV-specific markers at approximately 22 kDa, 27 kDa, 50 kDa, and 95 kDa, corresponding to CD81, CD9, CD63, and ALIX, which are indicative of vesicles of endosomal origin (Figure 1A). Importantly, bands of 45 and 90 kDa, corresponding to β-actin and calnexin, were absent in all EV extracts, excluding potential contamination with intracellular organelles or cell debris (Figure 1A). In addition, both EVs and parental moDCs expressed the marker CD11b (170 kDA), while CD14 (60 kDa) expression was restricted to monocytes and absent in EVs, confirming that the vesicles originated from differentiated moDCs rather than residual undifferentiated precursors (Figure 1B,C).

As observed by NTA, the mode size of the vesicles derived from allergic patients and control samples was comparable (165.10 ± 7.47 nm and 171.43 ± 5.21 nm, respectively). Although the size distributions displayed several peaks, the predominant vesicle population in both groups centered around ~170 nm, indicating a broadly similar size profile (Figure 1D). Also, the concentration of isolated EVs was determined using NTA. Three samples from each study group were analyzed, and the mean concentration of EVs released was reported. We observed that the EV concentration was 3.56 × 10^9^ ± 9.37 × 10^8^ particles/mL in non-allergic controls and 4.33 × 10^9^ ± 8.63 × 10^8^ particles/mL in CMAIE patients (Figure 1E). It is important to note that no significant differences were observed in EV secretion between non-allergic controls and CMAIE children, indicating that both groups released comparable amounts of EVs.

Together, these findings support the identity, integrity, and cellular origin of the isolated vesicles.

### 2.3. EVs from Allergic Patients Induce Eosinophil ROS Production and Migratory Response

To develop these assays, CMAIE-derived EVs were incubated with heterologous eosinophils obtained from allergic children, owing to time delays in EV isolation and the limited survival of eosinophils.

**Figure 2:**
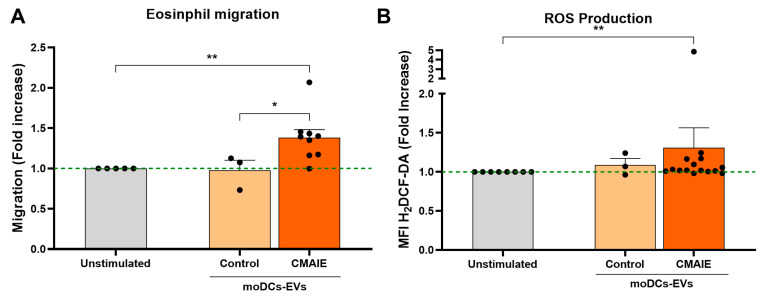
**Extracellular vesicles from CMAIE patients enhance eosinophil migration and ROS production.** (**A**) Migration of eosinophils unstimulated or stimulated with CMAIE patients or control-derived EVs. Unstimulated: 15 μL of free EVs-PBS 1X/EDTA 5 mM were added. (**B**) Production of ROS by eosinophils unstimulated or stimulated with EVs from CMAIE patients or control individuals. Unstimulated: 15 μL of ultracentrifuged PBS 1X/EDTA 5 mM were added. Statistical analyses were performed using the Wilcoxon rank-sum test. * *p* < 0.05, ** *p* < 0.01. CMAIE: cow’s milk allergy IgE-mediated, EVs: extracellular vesicles, moDCs: monocyte-derived dendritic cells.

Functional assays revealed that CMAIE-derived EVs exerted an activating effect on eosinophils. In migration assays, eosinophils exposed to EVs from allergic patients exhibited significantly enhanced migration compared with unstimulated controls, whereas EVs obtained from non-allergic donors did not elicit any detectable response (1.38 ± 0.09-fold increase; *p* < 0.01, vs. 0.98 ± 0.12-fold increase; *p* > 0.05, Figure 2A).

Similarly, exposure to CMAIE-derived EVs significantly increased intracellular ROS generation in eosinophils, a hallmark of eosinophil activation associated with degranulation and pro-inflammatory signaling. In contrast, eosinophils treated with EVs from non-allergic donors showed no change in ROS production relative to baseline levels (1.31 ± 0.25-fold increase; *p* < 0.01, vs. 1.09 ± 0.08-fold increase; *p* > 0.05, Figure 2B).

Together, these findings demonstrate that EVs released by moDCs from allergic individuals could enhance eosinophil ROS production and migratory behavior. This functional activity may contribute to the recruitment and activation of eosinophils in target tissues, thereby amplifying the pro-inflammatory microenvironment, which could be a novel mechanism in the progression of CMAIE.

### 2.4. Allergic Patient-Derived EVs Modulate T1 and T2 Responses in Lymphocytes

**Figure 3:**
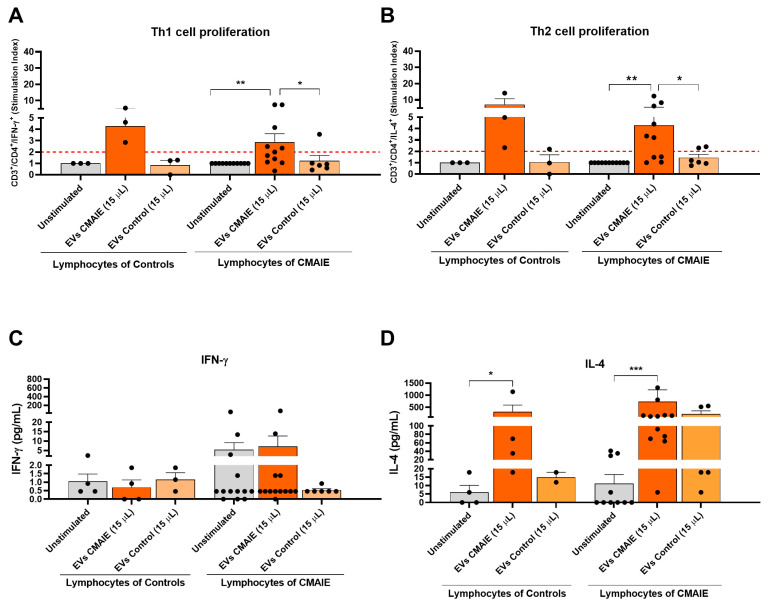
**EVs from CMAIE patients induce a mixed Th1/Th2 proliferative response and skew cytokine secretion toward Th2.** CD14^−^ PBMCs were cultured with either EV-free medium alone (unstimulated), EVs from CMAIE patients or EVs from non-allergic donors. Proliferation was quantified using flow cytometry, and T1 (**A**) and T2 (**B**) responses were evaluated via intracellular staining of IFN-γ and IL-4, respectively. A stimulation index (SI) > 2 was considered biologically significant (dashed red line indicates the threshold above which the stimulation index is considered positive). (**C**) IFN-γ levels (pg/mL) were not significantly upregulated in supernatants from CD14^−^ PBMCs stimulated with EVs. (**D**) IL-4 levels (pg/mL) were significantly upregulated in CD14^−^ PBMCs from both controls and patients when exposed to CMAIE-derived EVs. Unstimulated: 15 μL of ultracentrifuged PBS 1X/EDTA 5 mM were added. * *p* < 0.05, ** *p* < 0.01, *** *p* < 0.001. EVs: extracellular vesicles.

The potential immunomodulatory effects of EVs from patients and non-allergic controls on lymphocyte proliferation were evaluated. In CD14^−^ PBMCs isolated from allergic patients, EV exposure induced a significant expansion of both IFN-γ^+^ (Th1) and IL-4^+^ (Th2) CD3^+^/CD4^+^ T-cell subsets, with SI exceeding the activation threshold of 2 (2.86 ± 0.74, *p* < 0.01 and 4.30 ± 1.17, *p* < 0.01, respectively, Figure 3A and 3B). Notably, EVs derived from CMAIE patients showed a trend toward enhancing the proliferation of Th1 and Th2 cells in cultures from non-allergic donors (4.30 ± 0.75 and 7.15 ± 3.60; Figure 3A,B). However, no significant differences were observed, suggesting that allergic EVs could possess intrinsic immunostimulatory properties capable of promoting helper T-cell activation. In contrast, EVs obtained from non-allergic controls failed to elicit any proliferative effect (Th1 SI = 1.20 ± 0.50, *p* > 0.05, and Th2 SI = 1.40 ± 0.30, *p* > 0.05; Figure 3A,B).

To further investigate the functional impact of EVs on inflammatory responses, cytokine production was quantified in cell-free supernatants following EV stimulation. While EVs from allergic patients induced a significant proliferation of CD3^+^/CD4^+^/IFN-γ^+^ cells, no IFN-γ upregulation in cell culture supernatant from EV-stimulated CD14^−^ PBMCs was observed (7.14 ± 5.54 pg/mL, for CMAIE EVs vs. 5.34 ± 3.78 pg/mL, for unstimulated; Figure 3C). Among the cytokines assessed, only IL-4 was significantly upregulated in CD14^−^ PBMCs from non-allergic controls and patients exposed to CMAIE-derived EVs (316.40 ± 275.70 pg/mL for CMAIE EVs vs. 5.97 ± 4.21 pg/mL, for unstimulated, *p* < 0.05; Figure 3D; 737.54 ± 485.20 pg/mL for CMAIE EVs vs. 11.19 ± 5.36 pg/mL, for unstimulated, *p* < 0.001; Figure 3D). Nevertheless, EVs from non-allergic infants did not exert significant upregulation of IL-4 in controls or CMAIE patients (14.90 ± 2.94 pg/mL, for non-allergic control EVs vs. 5.97 ± 4.21 pg/mL for unstimulated; Figure 3D; 223.00 ± 128.20 pg/mL vs. 11.19 ± 5.36 pg/mL, for unstimulated; Figure 3D). This enhancement of IL-4 secretion is consistent with the observed expansion of CD3^+^/CD4^+^/IL-4^+^ T cells in previous assays and reinforces the notion that CMAIE-derived EVs could promote a Th2-skewed immune environment.

Together, these results demonstrate that EVs from CMAIE patients actively modulate T-cell proliferation, promoting the expansion of both Th1 and Th2 subsets. However, despite this mixed proliferative response, functional analysis revealed that only the Th2-associated cytokine IL-4 was significantly upregulated, with no corresponding increase in IFN-γ. These data support the evidence that EVs released by allergic patients selectively enhance Th2-associated cytokine production, thereby driving the specific amplification of IgE-mediated immune responses.

### 2.5. CMAIE Patient-Derived EVs Are Internalized by Eosinophils and PBMCs

**Figure 4:**
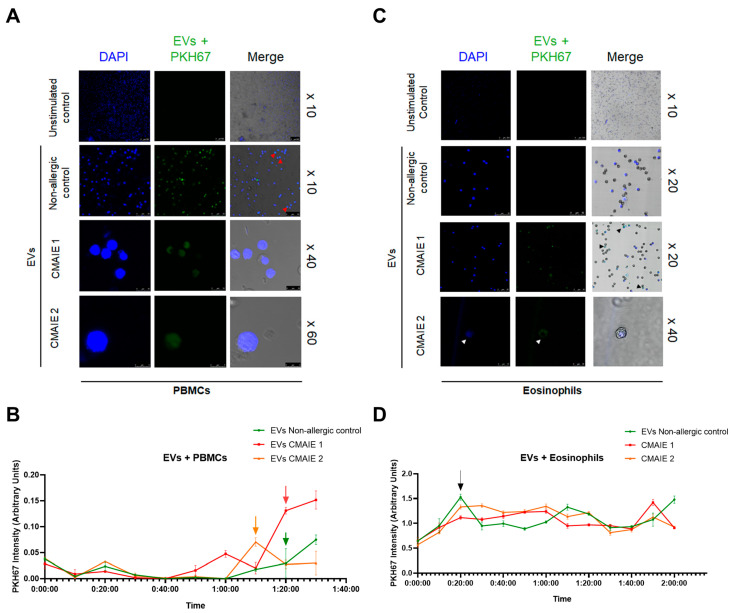
**Lymphocyte and eosinophil uptake of EVs from CMAIE patients and non-allergic controls.** (**A**) EV uptake by PBMCs. Red arrows depict the merge of the blue and green signals of lymphocytes and EVs, respectively. (**B**) In PBMCs, uptake remained comparable and low during the 0–60 min period, but showed a pronounced increase in CMAIE-derived EVs after 70-80 min (green, red and orange arrows). (**C**) EV uptake by eosinophils. Black arrows depict the merge of the blue and green signals of eosinophils and EVs, respectively. (**D**) In eosinophils, a rapid increase in uptake was observed in the first 0–20 min across all conditions (black arrow). CMAIE 1: Cow’s milk allergy IgE-mediated patient sample 1; CMAIE 2: Cow’s milk allergy IgE-mediated patient sample 2; EVs: extracellular vesicles.

To explore the cellular interactions of EVs released from moDCs of CMAIE patients and non-allergic controls, confocal microscopy was used to assess their uptake and localization in target immune cells. In these experiments, ~6.49 × 10^7^ particles from CMAIE patients and ~5.34 × 10^7^ particles from non-allergic controls were used. Fluorescently labeled EVs were efficiently internalized by both eosinophils and PBMCs. Interestingly, the intracellular distribution of EVs differed according to the target cell type. In PBMCs, EVs from both CMAIE and non-allergic infants were detected in the perinuclear region (Figure 4A). Nevertheless, in eosinophils, EVs from both patients and non-allergic controls accumulated predominantly in the cytoplasm (Figure 4B).

Moreover, EV internalization was evaluated to assess the time-dependent uptake of labeled vesicles in both cell types. As shown in Figure 4C, the uptake of EVs by PBMCs increased over time in all conditions. During the initial incubation period, 0–60 min, EV uptake remained low and comparable between EVs from controls and EVs from CMAIE samples. However, after approximately 80 min, EVs derived from CMAIE samples showed an increase in EV fluorescence intensity, indicating greater internalization by PBMCs. By 120 min, EVs from CMAIE exhibited the highest uptake, reaching a peak intensity of approximately 0.12 arbitrary units, whereas EVs from non-allergic donors displayed only a slight rise in fluorescence.

In contrast, eosinophils internalized EVs over time in all conditions (Figure 4D). During the initial phase, 0–20 min, a rapid increase in EVs fluorescence intensity was observed, indicating early uptake of EVs by eosinophils. This peak was similar across EVs from non-allergic donors and EVs from CMAIE patients, suggesting a comparable initial internalization. Following this early uptake phase, EV intensity remained relatively stable throughout the 2 h observation period, with no major differences between EV sources. These results show that eosinophils internalize EVs to a similar extent regardless of clinical condition.

These findings seem to indicate that EVs interact dynamically with immune effector cells and that their intracellular localization could be cell type-specific, supporting their role as selective mediators of immune modulation in allergic inflammation.

## 3. Discussion

This exploratory study provides novel evidence that EVs released by moDCs from patients with CMAIE actively participate in immune modulation by promoting lymphocyte proliferation and enhancing eosinophil migration and ROS production, suggesting novel cellular mechanisms in the progression and maintenance of CMA pathophysiology. We demonstrated that these vesicles are efficiently internalized by both PBMCs and eosinophils, exhibiting distinct intracellular localization patterns, which could suggest a novel cell type-specific mechanism of action (cytoplasmic accumulation in eosinophils, consistent with activation of cytoplasmic signaling cascades) and perinuclear localization in PBMCs, implying a potential role in the modulation of transcriptional activity [21,22,23]. Functionally, EVs derived from allergic patients induced a pronounced proliferative response in both Th1 and Th2 CD3^+^/CD4^+^ T-cell subsets, with an increase in IL-4 secretion, while concurrently enhancing eosinophil migration and ROS production. Collectively, these findings support the idea that CMAIE-derived EVs could serve as bioactive mediators capable of amplifying allergic inflammation through the coordinated activation of key effector cells involved in IgE-mediated responses.

The characterization of EVs confirmed the presence of the classical exosomal markers CD9, CD63, CD81, and Alix together with the absence of calnexin, thereby verifying the purity of the vesicular preparations. These findings are consistent with the established molecular signature of DC-derived EVs reported in previous studies [24]. In addition, the loss of CD14 expression and the maintenance of CD11b in both moDCs and their corresponding EVs further supported the successful differentiation of monocytes into DC and validated the cellular origin of the isolated vesicles [25,26]. Complementary NTA revealed a homogeneous population of particles within the expected size range for EVs [27]. These findings provide evidence of the purity of these EVs from moDCs.

At the lymphocyte level, EVs from CMAIE patients significantly enhanced the proliferation of both Th1 and Th2 cell subsets. Th2 proliferation is a hallmark of allergic reactions such as CMA, and previous studies have shown that DC-derived exosomes promote Th2 polarization of naïve T cells by displaying peptide-MHC complexes and expressing costimulatory molecules (CD40, CD80, CD86) [28]. Similarly, DC-derived exosomes have also been reported to promote Th1 polarization [29]. Th1 cells contribute to immune regulation by enhancing IgG production and suppressing IgE-mediated responses [30]. In our study, EVs from CMAIE patients induced comparable stimulation of both Th1 and Th2 lymphocytes, confirming their ability to promote Th2 activation typical of allergic responses. Furthermore, a Luminex-based cytokine analysis revealed a significant increase in IL-4 production in PBMCs from both CMAIE patients and control individuals following stimulation with EVs from CMAIE patients, whereas IFN-γ release did not increase in PBMCs following EV stimulation. This observation may reinforce our findings, indicating that CMAIE-derived EVs can promote a Th2-skewed immune response by enhancing IL-4 secretion, a hallmark cytokine of Th2 lymphocytes that plays a central role in the initiation and amplification of allergic inflammation [31]. In addition, this selective cytokine induction suggests that these EVs derived from allergic patients may contribute to an imbalance in T1/T2 responses, favoring T2 polarization and thereby potentially exacerbating allergic inflammation. The concurrent increase in Th1 lymphocyte proliferation could be attributed to the heterogeneity of EV cargo among patients, suggesting that not all CMAIE-derived EVs are strictly pro-allergenic; some may carry molecules with more tolerogenic properties, potentially explaining the dual activation pattern [30], although further studies are needed to confirm this. Remarkably, CMAIE-derived EVs also may have induced the proliferation of lymphocytes from non-allergic donors, while EVs from non-allergic individuals showed no effect. This finding could indicate that EVs from allergic patients may carry transferable pro-inflammatory signals capable of promoting a pro-allergic environment even in immune cells from non-allergic individuals. In line with previous studies, EVs from allergic subjects appear to drive a Th2-skewed immune response in non-allergic recipients, potentially increasing their susceptibility to developing allergic reactions [15].

On the other hand, eosinophils, which are recognized contributors to allergic inflammation, also responded to CMAIE-derived EVs. Functional assays revealed that these EVs enhanced both eosinophil migration and ROS production. The increased migratory response may suggest the presence of chemotactic molecules within the EV cargo, potentially cytokines, chemokines, or microRNAs capable of modulating, among others, CCR3 signaling, as previously described in asthmatic models [32,33]. This chemotactic activity may facilitate eosinophil recruitment to inflammatory sites, including the esophageal mucosa, where cow’s milk proteins can elicit eosinophilic esophagitis [34]. The observed increase in ROS production further supports the role of EVs in eosinophil activation. While physiological levels of ROS are necessary for immune defense and homeostasis, excessive ROS contributes to oxidative stress, epithelial barrier damage, and tissue remodeling, pathophysiological hallmarks of allergic inflammation [35]. These findings imply that DC-derived EVs may amplify eosinophil-mediated tissue injury, worsening the inflammatory state of an allergic reaction [36]. Altogether, these results support the notion that EVs from DCs are potent mediators of intercellular communication in CMA. Their ability to induce lymphocyte proliferation and eosinophil activation underscores a dual role in sustaining allergic inflammation and possibly promoting disease progression.

In addition, a confocal microscopy-based uptake assay was performed to evaluate the interaction of EVs from CMAIE patients with PBMCs and eosinophils. EVs were successfully internalized in both cell types but showed distinct intracellular distributions. In PBMCs, EVs accumulated in the perinuclear region, suggesting potential modulation through nuclear signaling pathways. Conversely, in eosinophils, EVs localized mainly in the peripheral cytoplasmic region, remaining outside the nucleus, implying cytoplasmic or organelle-related activity. These findings align with previous studies showing that DC-derived exosomes interact differently with immune cells. However, in other studies, it was found that DC-derived exosomes were internalized by monocytes but remain on the surface of B and T cells [37], whereas in this study, it was found that EVs from allergic patients accumulated in the perinuclear region of PBMCs. On the other hand, exosomes from asthmatic eosinophils have been reported to localize in the perinuclear region without entering the nucleus [33]. Additionally, according to the kinetics of internalization, we observed that EVs were internalized more rapidly in eosinophils than in PBMCs. Eosinophils can internalize extracellular particles more efficiently than lymphocytes due to their expression of a broad repertoire of innate immune receptors, including Fc receptors that recognize antibody-opsonized targets, enabling receptor-mediated phagocytosis and pinocytosis [38]. In contrast, lymphocytes primarily utilize highly antigen-specific receptors that mediate endocytosis of cognate antigens but are not specialized for broad, nonspecific particle uptake.

Beyond the cellular and immunological mechanisms described herein, it is imperative to contextualize these findings within the nutritional management of CMA. Cow’s milk represents a fundamental source of proteins, highly bioavailable calcium, and vitamins essential for infant growth and bone development [39]. Consequently, the strict avoidance diet, currently the gold standard for CMA management, poses significant challenges, including the risk of growth faltering and specific nutrient deficiencies if not properly managed [40]. While eHF and AAF are effective clinical alternatives, they differ biologically from the complex matrix of whole milk and are often associated with higher costs and palatability issues [4]. Furthermore, the rising popularity of plant-based beverages as alternatives for allergic patients warrants caution. Unlike standardized medical formulas, many plant-based substitutes (e.g., rice, almond, or soy drinks) may lack the protein density and micronutrient profile required for infants and young children, potentially leading to nutritional imbalances unless adequately fortified [6]. Therefore, understanding the EV-mediated inflammatory pathways identified in this study is not merely of scientific interest; it is a crucial step toward developing immunotherapeutic strategies (such as oral immunotherapy or other novel therapies) that can restore tolerance. Restoring the ability to consume cow’s milk would not only resolve allergic inflammation but also ensure that the patient receives the optimal nutritional benefits of dairy, avoiding the limitations of long-term exclusion diets. An alternative approach emerges from the results of this study; therefore, targeting EV biogenesis may represent a promising strategy for the management of cow’s milk allergy. Inhibition of EV release could modulate T-cell and eosinophil responses, highlighting the potential of EV-based interventions in CMA.

Despite the promising results, we are aware that this study has several limitations that should be acknowledged, most notably, the very young age of the infants and the challenges associated with obtaining biological samples. The sample size, especially of non-allergic controls, was relatively small, which limited the ability to perform detailed subgroup analyses and decreased the generalizability of the comparative analyses. Furthermore, the restricted sample volume constrained the number of parallel assays and the breadth of vesicular characterization (e.g., shape characterization by electron microscopy and EV cargo analysis). Another important limitation is the absence of oral food challenge (OFC) testing to confirm the diagnosis. For ethical and safety reasons, OFC was not performed in this pediatric cohort, and the diagnosis was based on a combination of a compatible clinical history and evidence of serum-specific IgE sensitization and/or positive skin prick tests (SPT). Nevertheless, this limitation should be considered when interpreting the results, as the lack of a confirmatory test could carry a risk of misclassification in some patients. Therefore, future studies involving larger, multicenter cohorts are warranted to validate these findings, expand the scope of functional analyses, and enhance their translational potential. Moreover, future research should focus on deciphering the molecular cargo of CMA-derived EVs, including their protein, lipid, and nucleic acid (e.g., miRNA) components, to identify the specific mediators responsible for lymphocyte proliferation, ROS production, and eosinophil migration. Comparative proteomic and transcriptomic analyses between EVs from allergic and non-allergic individuals could reveal novel biomarkers and therapeutic targets. Understanding how DC-derived EVs influence the immune microenvironment will be crucial for developing strategies to modulate EV-mediated signaling, potentially offering innovative interventions to restore immune tolerance in food allergies.

## 4. Materials and Methods

### 4.1. Study Design and Participants

A prospective study was conducted to evaluate immunological differences between infants diagnosed with CMAIE (*n* = 16) and non-allergic/non-atopic controls (*n* = 3). The cohort included infants aged 0–12 months who were referred to the Pediatric Allergology and Gastroenterology units at Fundación Jiménez Díaz Hospital.

The CMAIE group comprised infants who exhibited clinical symptoms consistent with IgE-mediated hypersensitivity reactions occurring within two hours of ingesting cow’s milk protein. Diagnostic confirmation was based on skin prick testing using pasteurized cow’s milk, with a positive response defined as a wheal diameter exceeding 3 mm or larger than the histamine control. Given the alignment between clinical presentation and test reactivity, controlled oral food challenges were not required for diagnosis in this subgroup [41]. The control group included infants without atopy or allergic disorders. These participants were selected during the same recruitment period while undergoing routine evaluations for unrelated conditions such as congenital infection risk. They presented no gastrointestinal or allergic symptoms and demonstrated negative results for allergen sensitization, making them suitable reference subjects for comparative analysis.

Ethical approval for the study was granted by the Fundación Jiménez Díaz Ethics Committee (Madrid, Spain) (approval number PIC 199-21_FJD), and all procedures were conducted in accordance with the principles of the Declaration of Helsinki. Written informed consent was obtained from the legal guardians of all enrolled participants.

### 4.2. Sample Collection and Cell Isolation

Peripheral blood samples were collected in tubes containing ethylenediaminetetraacetic acid (EDTA) as an anticoagulant (BD Vacutainer^®^, Becton Dickinson, Franklin Lakes, NJ, USA). Peripheral blood mononuclear cells (PBMCs) were isolated from all infants recruited in the study. Before blood fractionation, the samples were diluted 1:1 with 0.9% saline solution (Braun, Melsungen, Germany). In the first step, different cellular fractions were separated by density gradient centrifugation using a lymphocyte isolation solution (Lymphoprep, Commercial Rafer SL, Zaragoza, Spain). A 5:3 ratio of diluted blood to Lymphoprep was used. The mononuclear cell fraction was washed twice with 0.9% saline solution (Braun, Melsungen, Germany) and resuspended in freezing medium composed of RPMI-1640 (Lonza, Basel, Switzerland) with 10% dimethyl sulfoxide (DMSO) (Sigma-Aldrich, Merck KGaA, Darmstadt, Germany). The cells were stored at −80 °C for 24–48 h before being transferred to liquid nitrogen for long-term storage.

Additionally, the eosinophil purification process continued with the removal of erythrocytes from the polymorphonuclear fraction using a lysis solution (NH_4_Cl 155 mM, KHCO_3_ 10 mM, EDTA 0.1 mM, pH 7.5). In the second step, residual cells from the polymorphonuclear fraction were discarded using the EasySep^TM^ Human Eosinophil Isolation Kit (StemCell Technologies, Vancouver, BC, Canada), following the manufacturer’s instructions. Eosinophils were used for functional assays.

### 4.3. Monocyte Isolation and Differentiation into Monocyte-Derived Dendritic Cells

Monocytes from all non-allergic and CMAIE infants recruited to the study were isolated from PBMCs using the EasySep^TM^ Human CD14 Positive Selection Kit II (StemCell Technologies, Vancouver, BC, Canada), following the instructions provided by the manufacturer. The remaining CD14^−^ cell fraction, including T lymphocytes, was stored at −80 °C for subsequent experiments.

After the purification of CD14^+^ monocytes from PBMCs obtained individually from each non-allergic control and CMAIE participant, the cells were differentiated into moDCs. Due to variations in the number of monocytes isolated between individual PBMC samples, 1 × 10^6^ cells were cultured in 1 mL of RPMI medium (Gibco, Thermo Scientific, Waltham, MA, USA) per well in 12-well plates, supplemented with 10% FBS without EVs (depleted by ultracentrifugation at 100,000× *g* at 4 °C for 22 h), 100 U/mL penicillin, 100 µg/mL streptomycin, 1 nmol/L sodium pyruvate, and 2 nmol/L L-glutamine. MoDC differentiation was carried out using interleukin 4 (IL-4) and granulocyte-monocyte colony-stimulant factor (GM-CSF) (PeproTech, Thermo Scientific, Waltham, MA, USA) with concentrations of 10 ng/mL and 20 ng/mL, respectively, adding them once every day for 7 days.

### 4.4. Isolation of EVs

EVs produced by 10^6^ moDCs from all non-allergic individuals and CMAIE patients were isolated from the supernatant of the culture medium. Then, it was subjected to a series of centrifugations in sequential steps to remove any cells and/or cellular debris (twice at 900 g for 4 min, twice at 2400 g for 4 min, and twice at 11,600 g for 1 min at room temperature). Subsequently, the supernatant was ultracentrifuged at 100,000× *g* for 1 h and 15 min at 4 °C with an Optima XPN-100 Ultracentrifuge (Beckman Coulter, Brea, CA, USA). The resulting EV pellet was washed once with 1 mL of free EVs-PBS 1X and ultracentrifuged again according to the conditions described above. Then, the pellet was resuspended in 60 μL of free EVs-PBS 1X/EDTA 5 mM. All functional experiments were carried out with 15 μL of EVs, equivalent to ~6.49 × 10^7^ particles from CMAIE patients and ~5.34 × 10^7^ particles for controls. After this, they were aliquoted and stored at −80 °C for later use in different assays. Some of them were used directly to obtain protein lysate.

### 4.5. Molecular and Size Characterization of EVs

To evaluate and characterize isolated EVs, protein extracts were obtained from EVs, monocytes, and moDCs isolated from PBMCs. Protein extraction was performed using a lysis buffer containing RIPA (Thermo Fisher Scientific, Waltham, MA, USA), PMSF 1X and Protein Inhibitor Cocktail 1 mM. After lysis, the samples were centrifuged and the supernatants were stored at −20 °C until use.

Protein concentration was measured using the Pierce BCA assay (Thermo Fisher Scientific, Waltham, MA, USA), and quantification was performed with an Implen NanoPhotometerTM N60 (Thermo Fisher Scientific, Waltham, MA, USA). Five μg of protein extracts were separated on 12% SDS-PAGE gels and transferred to PVDF membranes. Membranes were blocked with PBS 1X-Tween 0.2%-nonfat dried milk 5% and incubated with primary (mouse anti-human calnexin, CD63, CD81, Alix, CD9, CD11b and CD14; Cell Signaling Technology, Danvers, MA, USA) and secondary antibodies (goat anti-mouse IgG antibody-HRP conjugate; Cell Signaling Technology, Danvers, MA, USA) diluted in PBS 1X-Tween 0.2% with nonfat dried milk 0.5%. Detection of target proteins was carried out by chemiluminescence using ECL reagent (Amersham, GE Healthcare Life Sciences, Marlborough, MA, USA), enabling the visualization of specific protein bands indicative of EV markers (CD63, CD81, Alix, CD69). Bands were visualized in the Amersham Imager 600 (GE Healthcare Life Sciences, Marlborough, MA, USA) chemiluminescence sensor.

For nanoparticle tracking analysis (NTA), a NanoSight NS300 (Malvern Panalytical, Malvern, Worcestershire, UK) was used to determine size and EV concentration. Samples were diluted in PBS 1X between 1:100 and 1:200 to achieve a particle concentration of 10^7^–10^9^ particles per milliliter. The camera focus was adjusted to ensure that the particles appeared as well-defined dots. Using the script control function, three 60 s videos were recorded for each sample, with automatic sample advancement and a 5 s interval between recordings.

### 4.6. Cellular Uptake of EVs

To assess the interaction of EVs with eosinophils and PBMCs, a confocal microscopy-based uptake assay was performed. EVs were isolated from moDCs of CMAIE patients and non-allergic controls and subsequently labeled with the fluorescent dye PKH67 (Sigma-Aldrich, Merck KGaA, Darmstadt, Germany), following the manufacturer’s protocol. A negative control was prepared using PBS 1X/EDTA 5 mM without EVs, processed identically to the experimental samples. Prior to EV addition, eosinophils and PBMCs were stained with 300 nM DAPI (Molecular Probes, Thermo Fisher Scientific, Waltham, MA, USA) for nuclear visualization. Fifteen μL of EVs were then added to cultures of eosinophils and PBMCs, similar to the experimental conditions used in the test. The uptake of the labelled EVs was assessed using a Leica TCS SP5 confocal microscope (Leica Microsystems, Wetzlar, Germany). Image acquisition, carried out every 8–10 min, was performed with a 10× magnification.

### 4.7. Functional Assays on Eosinophils

Isolated eosinophils were used after being cultured for 24 h with IL-5 (5 ng/mL) and GM-CSF (10 ng/mL). Migration assays were performed using 24-well plates (Corning^®^, Corning, NY, USA) with cell culture transwells containing a 5 μm pore size (Merck Millipore, Merck KGaA, Darmstadt, Germany). A total of 2.5 × 10^5^ eosinophils obtained from the previously described culture conditions were added to the upper chamber of the transwell. The lower wells were filled with 600 μL of complete RPMI-1640 medium with 15 μL of EVs and without EVs. Eotaxin (100 ng/mL) (R&D Systems, Minneapolis, MN, USA) was used as a positive control for the chemoattraction of eosinophils. Migration was carried out by incubating the cells at 37 °C in a 5% CO_2_ atmosphere for 120 min. After the incubation period, the number of migrated eosinophils was determined by quantifying the number of events over a 3 min period using flow cytometry (FACSCanto II, BD Biosciences, Franklin Lakes, NJ, USA) at a medium flow rate. An ultracentrifuged medium without EVs was used as a negative control.

In addition, to detect and quantify ROS, 2.5 × 10^5^ eosinophils from allergic patients were cultured per well in 24-well plates (Corning^®^, Corning, NY, USA) for 24 h with IL-5 (5 ng/mL) and GM-CSF (10 ng/mL) (R&D Systems, Minneapolis, MN, USA). Subsequently, EVs from CMAIE patients, 15 μL of EVs from non-allergic individuals, or free EVs-PBS 1X/EDTA 5 mM (control) were added and incubated for 2 h at 37 °C in a 5% CO_2_ atmosphere. After incubation, the intracellular fluorescent probe used to detect ROS, 2′,7′-dichlorodihydrofluorescein diacetate (H_2_DCF-DA, Thermo Fisher Scientific, Waltham, MA, USA), was added at a final concentration of 5 μM. The cells were then incubated for 30 min at 37 °C in a 5% CO_2_ atmosphere, and ROS production was evaluated using medium fluorescence intensity values (MFI) by flow cytometry with a FACSCanto II cytometer (BD Biosciences, Franklin Lakes, NJ, USA). A total of 20,000 events were analyzed in each assay. Pyocyanin (250 μM) (Abcam, Cambridge, UK) was used as the positive control for ROS production.

### 4.8. Proliferation and Cytokine Determination on Lymphocytes

Two hundred thousand CD14^−^ PBMCs were seeded in a round-bottom 96-well plate (200,000 cells/well) (Corning^®^, Corning, NY, USA), and 15 μL of EVs from CMAIE or non-allergic individuals were added and incubated for 72 h at 37 °C in a 5% CO_2_ atmosphere. After, the specific proliferation of different lymphocyte subpopulations (Th1: CD3^+^/CD4^+^/INF-γ^+^ and Th2: CD3^+^/CD4^+^/IL-4^+^) was evaluated by 5,6-carboxyfluorescein diacetate N-succinimidyl ester (CFSE) dilution assay, analyzing the expression of the CFSE^dim^ cells by flow cytometry. Also, unstimulated CD14^−^ PBMCs were used as a negative control, and PHA (Sigma-Aldrich, Merck KGaA, Darmstadt, Germany) was added at 12 μg/mL to the positive control for proliferation. Results were expressed as the stimulation index (SI), which was calculated for each cell subset as [%CFSE^low^ EVs stimulated lymphocytes/%CFSE^low^ unstimulated lymphocytes].

The supernatants from the CD14^−^ PBMC culture were collected, and cytokine production was determined using the human MILLIPLEX^®^ Multiplex Assays Using Luminex^®^ Technology (Merck KGaA, Darmstadt, Germany), analyzing these cytokines: EGF, FGF2, GM-CSF, IFNA2, IFNG, IL10, IL12p70, IL13, CD40L, IL17A, IL9, IL1b, IL2, IL4, IL5, IL8, IP10, MCP1, MIP1a, MIP1b and VEGF.

### 4.9. Statistical Analysis

Data were expressed as the mean ± standard error of the mean (SEM) for parametric data, the median (25th–75th percentile) for non-parametric data, and percentages for categorical variables. The normality of continuous variables was assessed using the Shapiro–Wilk test. Categorical variables were compared using Fisher’s exact test. Continuous variables were compared using the Kruskal–Wallis test, with Dunn’s post-test correction for non-parametric data, or one-way ANOVA with Bonferroni correction for parametric data. Analyses were carried out using Graph-Pad Prism 8 (San Diego, CA, USA). *p*-values < 0.05 were considered statistically significant.

## 5. Conclusions

In conclusion, this exploratory study highlights a previously unrecognized immunoregulatory mechanism in CMA, whereby DC-derived EVs orchestrate cross-talk between lymphocytes and eosinophils to sustain allergic inflammation. These findings expand our understanding of the immunopathology of CMA and suggest that targeting EVs or their cargo may represent a promising avenue for future therapeutic development in allergic diseases.

## Figures and Tables

**Table 1:** Clinical characterization of CMAIE patients and controls.

	Control (*n* = 3)	CMAIE (*n* = 16)	*p*-Value
**Demographic data**			
Males, *n* (%)	3 (100)	8 (50)	NS
Age (months) ^†^	5 (3–5)	7 (5.25–8)	NS
**Symptoms**			
Gastrointestinal, *n* (%)	0 (0)	6 (37)	NS
Respiratory, *n* (%)	0 (0)	2 (12)	NS
Urticaria, *n* (%)	0 (0)	16 (100)	***
**Skin prick test positivity, *n* (%)**	0 (0)	16 (100)	**
**Specific IgE (kU/L)**	T_0_	T_0_	
sIgE milk ^†^	0.01 (0–0.2)	2.53 (0.85–10.51)	*
Positive sIgE milk, *n* (%)	0 (0)	14 (87.5)	*
sIgE α-lactalbumin ^†^	0 (0–0.1)	1.29 (0.02–3.02)	*
Positive sIgE α-lactalbumin, *n* (%)	0 (0)	9 (56.25)	NS
sIgE β-lactoglobulin ^†^	0.01 (0.01–0.23)	0.59 (0.04–2.47)	*
Positive sIgE β-lactoglobulin, *n* (%)	0 (0)	11 (68.75)	NS
sIgE casein ^†^	0.01 (0–0.01)	0.31 (0.03–4.1)	NS
Positive sIgE casein, *n* (%)	0 (0)	8 (50.0)	NS
**IgG Milk (kU/L)**	0.18 (0.15–4.88)	0.31 (0.15–2.26)	NS

NS: Non-significant, ^†^: 25th–75th percentile, CMAIE: cow’s milk allergy IgE-mediated. * *p* < 0.05, ** *p* < 0.01, *** *p* < 0.001.

## Data Availability

The data that support the findings of this study are available on request from the corresponding author. The data are not publicly available due to privacy or ethical restrictions.
